# Dysregulated miR-155 and miR-125b Are Related to Impaired B-cell Responses in Down Syndrome

**DOI:** 10.3389/fimmu.2018.02683

**Published:** 2018-11-20

**Authors:** Chiara Farroni, Emiliano Marasco, Valentina Marcellini, Ezio Giorda, Diletta Valentini, Stefania Petrini, Valentina D'Oria, Marco Pezzullo, Simona Cascioli, Marco Scarsella, Alberto G. Ugazio, Giovanni C. De Vincentiis, Ola Grimsholm, Rita Carsetti

**Affiliations:** ^1^B cell Pathophysiology Unit, Immunology Research Area, Bambino Gesù Children's Hospital, IRCCS, Rome, Italy; ^2^Division of Rheumatology, Bambino Gesù Children's Hospital, IRCCS, Rome, Italy; ^3^Research Laboratories, Bambino Gesù Children's Hospital, IRCCS, Rome, Italy; ^4^Pediatric and Infectious Disease Unit, Bambino Gesù Children's Hospital, IRCCS, Rome, Italy; ^5^Institute of Child and Adolescent Health, Bambino Gesù Children's Hospital, IRCCS, Rome, Italy; ^6^Unit of Otolaryngology, Bambino Gesù Children's Hospital, IRCCS, Rome, Italy; ^7^Department of Rheumatology and Inflammation Research, University of Gothenburg, Gothenburg, Sweden; ^8^Unit of Diagnostic Immunology, Department of Laboratories, Bambino Gesù Children's Hospital, IRCCS, Rome, Italy

**Keywords:** Down Syndrome, B cell, miR-155, miR-125b, antagomiR, germinal center, plasma cells, immunodeficiency

## Abstract

Children with Down Syndrome (DS) suffer from immune deficiency with a severe reduction in switched memory B cells (MBCs) and poor response to vaccination. Chromosome 21 (HSA21) encodes two microRNAs (miRs), miR-125b, and miR-155, that regulate B-cell responses. We studied B- and T- cell subpopulations in tonsils of DS and age-matched healthy donors (HD) and found that the germinal center (GC) reaction was impaired in DS. GC size, numbers of GC B cells and Follicular Helper T cells (T_FH_) expressing BCL6 cells were severely reduced. The expression of miR-155 and miR-125b was increased in tonsillar memory B cells and miR-125b was also higher than expected in plasma cells (PCs). Activation-induced cytidine deaminase (AID) protein, a miR-155 target, was significantly reduced in MBCs of DS patients. Increased expression of miR-155 was also observed *in vitro*. MiR-155 was significantly overexpressed in PBMCs activated with CpG, whereas miR-125b was constitutively higher than normal. The increase of miR-155 and its functional consequences were blocked by antagomiRs *in vitro*. Our data show that the expression of HSA21-encoded miR-155 and miR-125b is altered in B cells of DS individuals both *in vivo* and *in vitro*. Because of HSA21-encoded miRs may play a role also in DS-associated dementia and leukemia, our study suggests that antagomiRs may represent pharmacological tools useful for the treatment of DS.

## Introduction

Down Syndrome (DS) [OMIM #190685] is the most frequent chromosomal disorder in humans, ranging from 1/300 to 1/1000 live births, and it is caused by an inherited extra copy of human chromosome 21 (HSA21) (1). Clinical features of DS include variable intellectual disability, a characteristic facial dysmorphism, cardiac, airway, and gastrointestinal anatomic anomalies, high risk of developing type I diabetes mellitus, celiac disease, acute leukemias as well as early onset Alzheimer's disease (1–4). Life expectancy has increased significantly in the past years, mainly because of effective surgical correction of cardiac malformations, increasing from 10 years in 1960 to 60–65 years nowadays (5). Immune deficiency is an integral feature of DS and infection-related mortality is still high. We and others have described alterations in the immune response that may play a substantial role in the development of recurrent infections, autoimmunity, and malignancy in DS (6–8). The T-cell compartment has been reported to be normal in DS, with the exception of the low frequency of naïve CD4^+^ T cells (6, 9, 10). On the other hand, all B-cell subpopulations are reduced in DS children. In the memory B cell (MBC) compartment, switched MBCs are 10-fold less than controls (6, 8). Furthermore, we showed that DS children respond poorly to primary immunization producing significantly less MBCs and antibodies (Abs) than their siblings (7). It is still not clear how the extra-copy of HSA21 causes the complex phenotype of DS. Bioinformatic studies have detected more than 500 genes, a high number of long non-coding RNA (lncRNA) and 14 microRNAs (miRs) encoded on HSA21 (11–15). It has been demonstrated that the presence of a third copy of HSA21 does not necessarily result in overexpression of HSA21-encoded genes (16), but rather in a complex dysregulation of chromatin function. This is demonstrated by the altered expression of genes that map on other chromosomes (11). miRs are small non-coding RNAs that modulate gene expression by binding to their target mRNAs, thus affecting their stability or preventing translation (17–19). Even though miRs constitute only 3% of the whole genome, each miR has hundreds of targets, thus modulating the expression of about 90% of all known genes (20). During cell development the expression of miRs is tightly regulated both at the transcriptional and the post-transcriptional level. Transcriptional regulation is lost when miR is translocated or ectopically expressed (21).

It has been demonstrated that among HSA21-encoded miRs, five are overexpressed in cells and tissues of DS patients (12, 22). Several studies have shown that miRs regulate immune functions. In particular, HSA21-derived miRs, miR-125b, and miR-155, play a role in the GC reaction and plasma cell (PC) differentiation (23, 24). In DS, the overexpression of miRs seems to be a direct consequence of the extra copy of HSA21 that contains the miRs in their normal chromosomal location. Thus, transcriptional regulation is maintained in DS.

Here, we investigated the role of HSA21-encoded miRs, miR-125b and miR-155, in the peripheral development and function of B cells of children with trisomy 21. We show that HSA21-derived miRs are overexpressed in B cells isolated from lymphoid tissues and peripheral blood of DS patients. The upregulation of miR-125b and miR-155 is mostly evident in MBCs, activated B cells, and plasma blasts (PBs)/PCs of DS patients, highlighting the crucial role of HSA21-derived miRs in the regulation of antigen-experienced B cells. Finally, we show that by blocking miR-155 *in vitro* we could partially reverse the abnormalities observed in MBCs and PBs of DS children. Thus, miR-125b and miR-155 are dysregulated in DS patients and are both crucial in coordinating human MBCs and PB biology.

## Materials and methods

### Study population

HD and DS patients were enrolled at Down Syndrome and Pediatric outpatient Clinic of Bambino Gesù Children's Hospital in Rome. The diagnosis of trisomy 21 was confirmed by karyotyping; patients carrying a Robertsonian translocation or chromosome 21 mosaicism were excluded. The study was approved by the Ethical Committee of Bambino Gesù Children Hospital, Rome.

### PBMCs and tonsils

Human peripheral blood mononuclear cells (PBMCs) from HD and children with DS were isolated on density gradient centrifugation (Lympholyte, CEDARLANE). Samples were frozen in heat inactivated fetal bovine serum (FBS, Hyclone Laboratories Logan UT) with 10% DMSO and stored in liquid nitrogen until further use. Tonsils obtained from HD and DS children undergoing routine tonsillectomy were processed into single cell suspension. Briefly, tonsillar mononuclear cells were extracted by mechanical disruption. The specimens were cut into fragments and mashed through a cell strainer. Next, ficoll density gradient centrifugation was performed (as above). The mononuclear cell layer was then collected and cells were frozen in FBS with 10% DMSO and stored in liquid nitrogen, as previously described. At the same time, part of fresh tonsil tissue was also sliced and snap frozen in liquid nitrogen for immunohistology.

### Stimulations and reagents

Cells were cultured at a concentration of 2.5 × 10^6^ cells/mL in 96-multiwell plates (Becton Dickinson, San Jose, CA, USA) and cultured for different time points as described in figure legends. CpG-B ODN2006 (Hycult Biotech) was used at 0.35 μM concentration. Complete medium was prepared as follows: RPMI-1640 (Gibco BRL, Life Technologies), 10% FBS, 1% L-Glutammine (Gibco BRL); 1% Antibiotics/Antimicotics (Gibco BRL), 1% sodium pyruvate (Gibco BRL).

### AntagomiR treatment

Lyophilized antagomiRs were custom synthesized according to Krutzfeldt et al. (25) (ThermoFisher) (Supplementary Figure S1B). Cells were washed twice in PBS, resuspended in serum-free medium, pre-incubated for 2 h at 37°C and supplemented with antagomiRs at a concentration of 2 μM (26). Cells were subsequently stimulated with CpG, as previously described, for seven days. The proportions of B cells and PCs were evaluated by flow cytometry. In parallel, after *in vitro* stimulation with CpG, cells were harvested and total RNA was extracted. By qPCR the expression level of silenced miRs was evaluated in comparison with scr-treated cells. Briefly, we calculated the relative level of miR expression in cells treated with antagomiRs. Then, miR levels were expressed as percentage of the scr-treated cells. In all experiments, the normalized level of miR in antagomiR-treated cells was roughly 10% of the level of the same miR in scr-treated cells. We calculated the percent of silencing by the following formula: scr-antagomiR treated cells. In our experiments, therefore the efficiency of silencing achieved was 100–10% = 90%.

### Flow cytometry

PBMCs and tonsil cells were stained with fluorochrome-conjugated Abs according to the standard operating procedure (see Supplementary Figure S1A for a complete list of Abs). B cell subsets were identified according to previous reports (27–29). The Cytofix/Cytoperm kit (BD Biosciences) was used for intracellular staining of BLIMP-1, AID, and BCL6 according to the manufacturer's recommendations. Dead cells were excluded from analysis by side/forward scatter gating. At least 100,000 gated events on living cells were analyzed, whenever possible, for each sample. Samples were acquired on a BD Fortessa X-20 (BD Biosciences).

### Cell sorting

Tonsil cells were washed and stained with fluorochrome-conjugated Abs. Tonsillar B-cell and T-cell subpopulations were sorted (Figures S2A,B). Sorting was performed using the FACSAria ™ III cell sorter (BD Biosciences). Post-sort purity was controlled for each sample and was higher than 98%.

### RNA extraction and real-time PCR analysis

Activated PBMCs from cultures and mononuclear cells from tonsils were lysed with Trizol (Trizol® Reagent, Applied Biosystem) and RNA was extracted according to manufacturer's instructions. Total RNA was retro-transcribed to cDNA using SuperScript® III Reverse Transcriptase (Invitrogen). For miRs, RNA was retro-transcribed to cDNA using Taq-Man MicroRNA Reverse Transcription Kit according to manufacturer's instructions. Quantitative PCR (qPCR) was performed using the 7900 HT Fast Real Time PCR System and commercial Taq-Man probes™ (Supplementary Figure S1B). Gene expression was normalized to ACTINβ or GAPDH and miRs expression was normalized to U6. Data are expressed as arbitrary units (AU) determined by using the 2^−Δct^ method, according to literature (30).

### Immunohistochemistry and immunofluorescence

Five micrometers tonsil sections were stained with Hematoxylin and Eosin. Images were acquired with a Leica DMi8 microscope. The whole section was reconstructed and a representative area (13.97 mm^2^) was used for analysis. For each patient and HD the number of GCs was counted and their area was calculated. The analysis was performed with the NIS-elements BR Imaging Software version 4.50. For immunofluorescence studies, sections from HD and DS were stained with fluorochrome-conjugated Abs against CD4, IgM, and IgD. CD4^+^ T cells were counted within the follicles and the cellular density (number of CD4^+^ T cells/GC area) was calculated for each section. Images were acquired with a Leica SP8X microscope.

### Statistical analysis

Data are presented as mean±SEM or median as described in figure legends. Statistical analysis was performed using the Prism software (GraphPad Prism 5, La Jolla, CA) and unpaired Student's *t*-test or One-way ANOVA Kruskal-Wallis test followed by Dunn's Multiple comparison test were used as described in figure legends. Data were considered statistically significant when *p* < 0.05.

## Results

### GCs and MBCs were decreased in tonsils of DS patients

**Figure 1:**
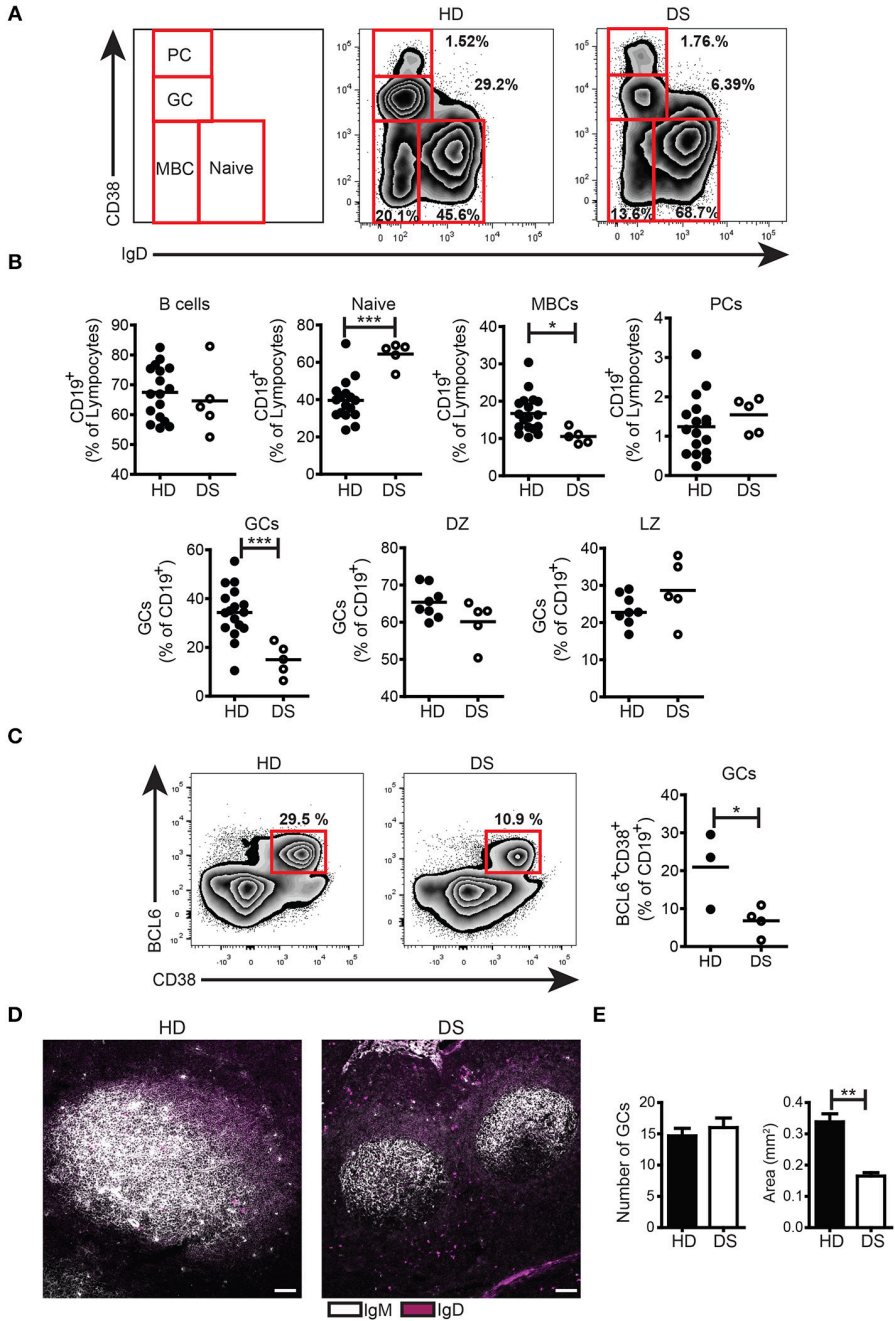
B cell populations and GCs in tonsils. **(A-C)** Flow cytometry analysis of B-cell subsets in tonsils stained with appropriate Abs. **(A)** Plots show the distribution and frequency of B-cell subsets in tonsils of a representative HD and DS. **(B)** Frequency of total B cells (expressed as percentage of lymphocytes), naïve, memory, plasma cells, GC (all expressed as percentage of CD19^+^), DZ, and LZ (all expressed as percentage of GCs) of HDs (*n* = 17, except DZ and LZ *n* = 8) and DS patients (*n* = 5, except DZ and LZ *n* = 3) is shown. **(C)** Representative plots and graph shows frequency of BCL6^+^ GC B cells in tonsils (HD *n* = 3; DS *n* = 4). Each dot represents a different HD or DS and black lines represent mean **(D)** Analysis of GC B cells in tonsils stained with IgM and IgD by immunofluorescence (IF). Images are 20X, scale bar 100 μm. **(E)** Bars show mean±SEM number (left) and area (mm^2^) (right) of GCs that were calculated on sections stained with H&E in HD (*n* = 3) and DS patients (*n* = 3). Data are representative of three independent experiments. Differences between groups determined by unpaired Student's *t*-test (^*^p = 0.05, ^**^p = 0.01, ^***^p = 0.001).

We collected tonsils from DS children and age-matched HD and analyzed the distribution of B-cell subpopulations in the two groups. B cells were gated as CD19^+^ and then divided into subpopulations according to the surface expression of CD38 and IgD following the Bm classification (27) (Figure 1A). The frequency of total B cells was similar between HD and DS children. Naïve B cells (Bm1-2, CD38^−^IgD^+^) were significantly increased in DS patients as compared to controls. MBCs (Bm5, CD38^−^IgD^−^CD27^+^) were instead significantly reduced in DS. When we separately analyzed IgM and switched MBCs, we found that switched MBCs were significantly less in DS tonsils (Supplementary Figure S3A), thus confirming previous observations in the peripheral blood (6, 8). GC B cells (Bm3-4, CD38^+^IgD^−^) were significantly reduced in DS children to about half of the normal numbers. The relative proportions of centroblasts (CXCR4^+^ CD86^−^) and centrocytes (CXCR4^−^ CD86^+^) (31, 32) were maintained. PCs identified either as CD38^++^IgD^−^ (Figures 1A,B) or CD38^++^CD27^++^ (Supplementary Figure S3B) were comparable between the two groups. In order to further characterize the reduction in GC B cells observed in DS children, we stained tonsil B cells for BCL6, a transcription factor that acts as master regulator of the GC response (33–35). BCL6 expression is tightly regulated during B-cell differentiation and is restricted to the GC where SHM and CSR occurs (36, 37). We found that GC B cells, expressing BCL6 (identified as CD38^+^IgD^−^BCL6^+^) were significantly reduced in DS patients compared to controls (Figure 1C). Thus, GC B cells, as well as MBCs, were reduced in DS tonsils, whereas the frequency of PCs was similar in patients and HD. The low frequency of GC B cells detected by flow cytometry could be explained either by a reduction in the number or size of GCs in DS patients. In order to clarify this important point, we identified B cell follicles and GCs by staining tonsil sections for IgM and IgD and analyzing them by confocal microscopy (Figure 1D) and H&E staining (Supplementary Figure S4). Both in HD and in DS, we counted the number and calculated the area of GCs. Although the number of GCs was the same in HD and DS patients, their size was significantly smaller in DS patients than in controls (Figure 1E). Thus, in the tonsils of DS children, GCs were small, containing fewer BCL6^+^ B cells, MBCs were reduced in number, whereas frequency of PCs was comparable between the two groups.

### T_FH_ cells are reduced in both tonsils and peripheral blood of DS patients

**Figure 2:**
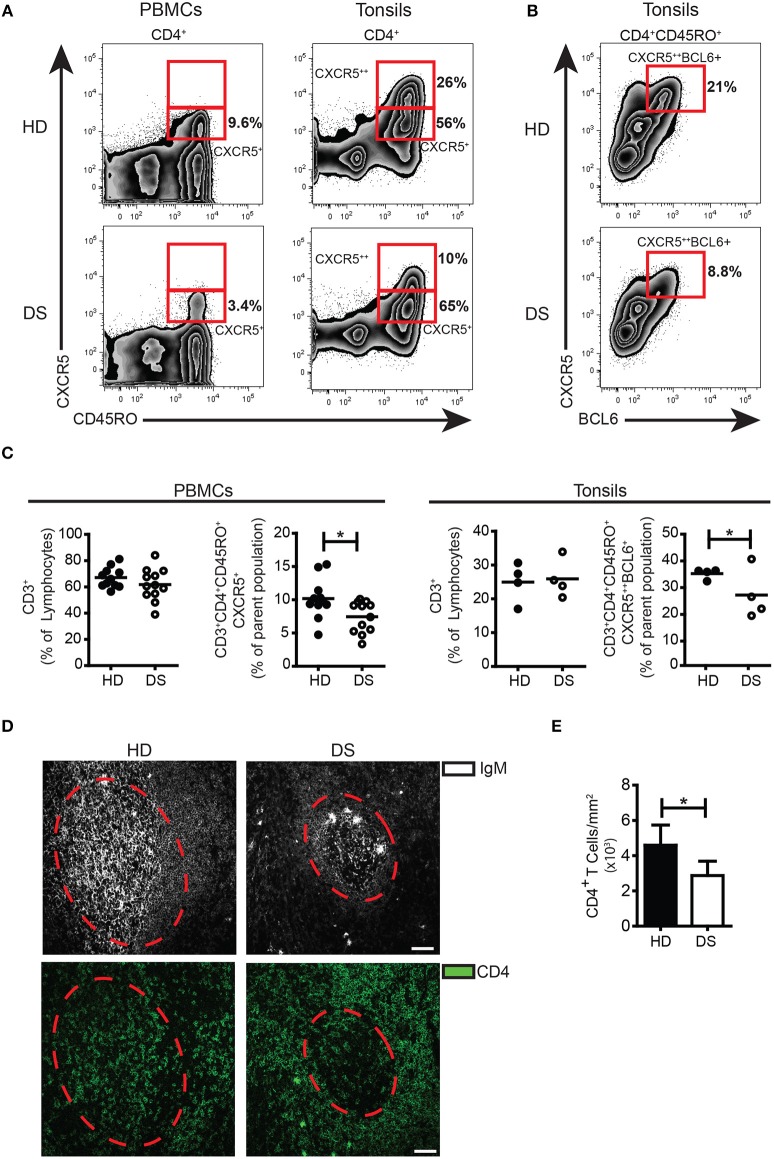
T_FH_ cells in tonsils and peripheral blood. **(A)** Plots show percentage of CD3^+^CD8^−^CD45RA^−^CD45RO^+^CXCR5^+^ and CXCR5^++^ in PBMCs and tonsils of a representative HD and DS patients. **(B)** Plots show percentage of CD3^+^CD4^+^CD45RA^−^CXCR5^++^BCL6^+^ cells in tonsils of a representative HD and DS patients. **(C)** Graphs show percentage of total CD3^+^ T cells in PBMCs and tonsils, CXCR5^+^ T_FH_-like cells in PBMCs and CXCR5^++^BCL6^+^ T_FH_ cells in tonsils of a representative HD and DS patients. Each dot represents a different HD or DS, black lines represent mean (peripheral blood: HD *n* = 11, DS *n* = 12; tonsils: HD *n* = 4, DS *n* = 4). **(D)** IF analysis of tonsils from HD and DS using anti-CD4 and anti-IgM Abs to identify T cells within the GC. Dashed line mark the GC area. Images are 20X, scale bar 75 μm. **(E)** Bars show mean±SEM cellular density of CD4^+^ within each GC (HD *n* = 3, DS *n* = 3). Differences between groups determined by unpaired Student's *t*-test (*p = 0.05).

Inside the GC, T_FH_ cells are responsible for the selection of B cell clones bearing high affinity receptors, favoring isotype switching and differentiation into MBCs and PCs. In human tissues, bona fide T_FH_ cells were identified as CD3^+^CD4^+^CD45RO^+^CD45RA^−^CXCR5^++^BCL6^+^(38, 39). Memory T cells expressing modest amounts of CXCR5 and lacking BCL6 (CD3^+^CD4^+^CD45RO^+^CD45RA^−^CXCR5^+^BCL6^−^) were considered T_FH_ precursors, locating mainly outside the GC (40). In tonsils, the frequency of T_FH_ precursors was comparable between DS and HD (Figure 2A), whereas the frequency of T_FH_ cells was significantly reduced in DS tonsils (Figure 2B,C). T_FH_-like cells have been also described in peripheral blood (40, 41). We found that in peripheral blood of DS patients, T_FH_-like cells were also reduced (Figure 2A,C). Next, we studied the localization of T cells within lymphoid tissues of DS patients and HD. Sections of tonsils were stained with Abs against CD4 and IgM in order to visualize T and B cells and their reciprocal localization (Figure 2D). The number of CD4^+^ T cells within the GCs (expressed as number of cells divided by the area of GCs to correct for the differences in the areas of GCs) were reduced in tonsils of DS children in comparison to those of HD (Figure 2E). In conclusion, we found that in the tonsils of DS children T_FH_ cells are reduced as shown by the decrease of both CXCR5^++^BCL6^+^ T cells and CD4^+^ T cells within the GCs. Our results suggest that the number of T_FH_ cells, with a crucial role in sustaining a correct GC response, is reduced in DS patients.

### Expression of chromosome 21-derived miRS was increased in tonsillar B-cell subpopulations of DS patients and affects the expression of AID and BLIMP-1

**Figure 3:**
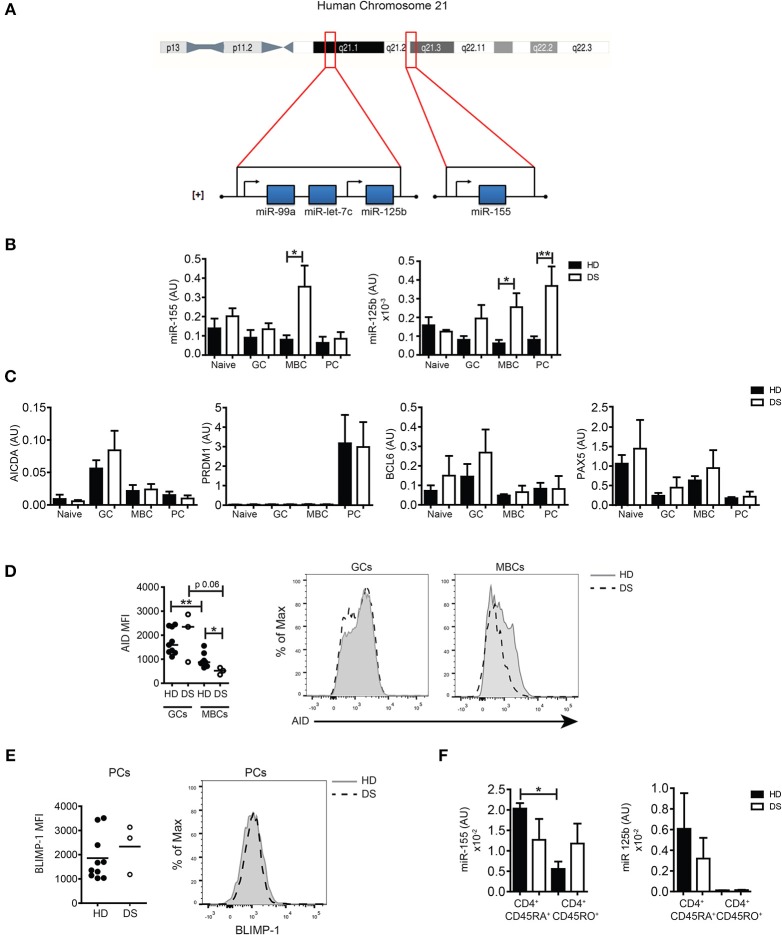
The expression of miRs in sorted tonsillar B cells. **(A)** Schematic Figure showing HSA21 and loci of miRs on the long arm of HSA21. **(B)** Bars show mean±SEM expression of miR-155 and miR-125b in sorted tonsillar B-cell populations (HD *n* = 4; DS *n* = 4). **(C)** Bars show mean mRNA expression±SEM of AICDA, PRDM1, PAX5, BCL6 in sorted tonsillar B-cell populations (HD *n* = 4; DS *n* = 4). **(D)** Graphs and histograms show flow cytometry analysis of AID expression in GCs and MBCs (HD *n* = 8; DS *n* = 3). **(E)** Graph and histogram show flow cytometry analysis of BLIMP-1 expression in PCs (HD *n* = 10; DS *n* = 3). Each dot represents a different HD or DS and black lines represent mean **(F)** Bars show mean±SEM expression of miR-125b and miR-155 expression in sorted CD4^+^ naive and memory T cells (HD *n* = 3; DS *n* = 3). Differences between groups determined by unpaired Student's *t*-test (*p = 0.05, **p = 0.01). In **(F)** one-way ANOVA Kruskal-Wallis test followed by Dunn's Multiple comparison test was performed (^*^p < 0.05).

HSA21 encodes miR-125b and miR-155, both important for B-cell biology. The miR-125b locus is in the subcentromeric region of the long arm of HSA21. Two additional miRs, let7c and 99a are included in the same cluster with 125b. Instead, miR-155 is located on a separate locus ~8,9 Mb downstream, toward the telomeric region ([+] strand, 5′-3′ direction) (Figure 3A). Both miR-155 and miR-125b play an important role in the control of the GC reaction. In order to investigate whether the expression of miRs was altered in the GC, we sorted B-cell populations from tonsils of HD and DS children (naïve B cells, GC B cells, MBCs, and PCs, according to the gating strategy shown in Supplementary Figure S2A). By qPCR, we evaluated the expression levels of the two miRs of interest and their target genes PRDM1 and AICDA, as well as BCL6 and PAX5. We found that in DS patients the expression of miR-155 was significantly higher in MBCs. The expression of miR-125b was increased in both MBCs and PCs (Figure 3B). AICDA mRNA was expressed in GC B cells, but no differences between HD and DS patients were noted. PRDM1 was highly expressed in PCs of both HD and DS patients. The mRNA for BCL6 was upregulated in GC B cells in HD and DS children (Figure 3C). As expected, PAX5 was mostly expressed in naïve and MBCs, with no significant differences between HD and DS patients. miRs regulate protein expression by two different mechanisms: inducing the degradation of mRNA and mainly inhibiting the translation process (18, 19). By flow cytometry we showed that the expression of AID, the protein encoded by AICDA, was comparable in the GC of both HD and DS children, but was significantly lower in MBCs of DS patients, mirroring the higher expression of miR-155 observed in these cells (Figure 3D). BLIMP-1 was equally expressed in tonsil-resident PCs of HD and DS (Figure 3E). Finally, we also analyzed the expression of miRs in sorted T-cell subpopulations. In tonsils of HD, miR-155 was downregulated in CD45RO^+^ T cells, whereas it was equally high in naïve and memory T cells of DS children. In tonsils, CD45RO^+^ T cells included all T_FH_ cells (Figure 3F). Our results show that the expression of miR-155 and miR-125b is altered in tonsillar B cells of DS patients: miR-155 was slightly increased in GC B cells and significantly over-expressed in MBCs, whereas expression of miR-125b was increased in PCs of DS children. Furthermore, miR-155 is downregulated in memory T cells, but remained high in memory T cells of DS children.

### HSA21-derived miRs were dysregulated in *in vitro* PBMCs of DS patients

**Figure 4:**
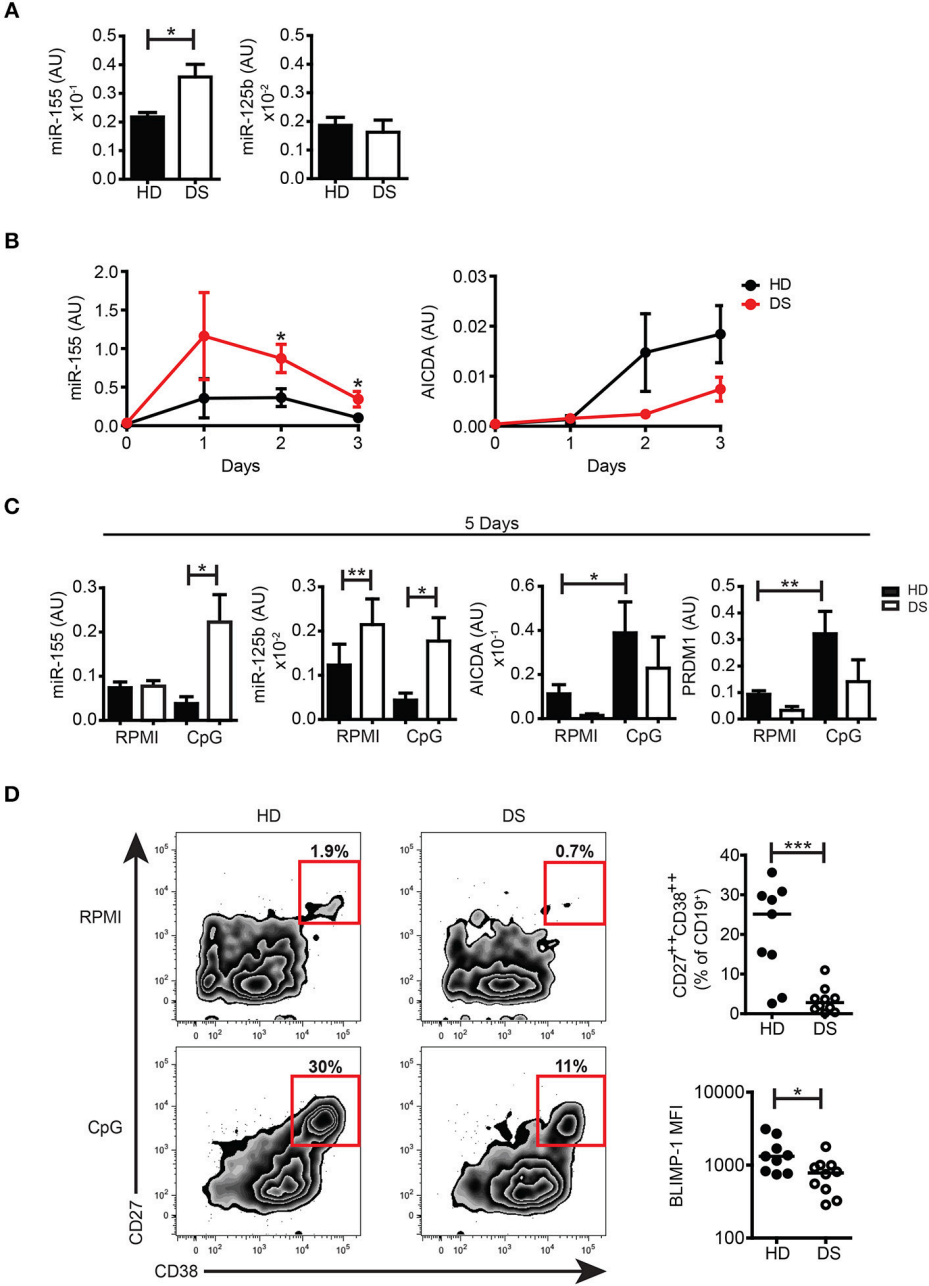
miRs expression in untreated cells and in *in vitro* activated PBMCs. **(A)** Bars show mean expression±SEM of miR-125b and miR-155 in untreated PBMCs from HD (*n* = 11) and DS children (*n* = 10). **(B)** Graphs show the mean expression±SEM of mRNA of miR-155 and AICDA in DS and HD at different time points (HD *n* = 6, DS *n* = 5). **(C)** Bars show mean expression±SEM of miR-125b and miR-155 in PBMCs stimulated with CpG for 5 days (HD *n* = 8; DS *n* = 4 pools, each pool is composed of 5 children) and mean expression±SEM of mRNA of AICDA and PRDM1 in PBMCs stimulated with CpG for 5 days (HD *n* = 8; DS *n* = 4 pools, each pool is composed of 5 children). **(D)** PBMCs were stimulated for 5 days with CpG and frequency of CD27^++^CD38^++^ plasma blasts was evaluated. Left panel shows representative flow cytometry plots of plasma blasts of a HD and a DS patient. Right panel shows frequency of plasma blasts and plasma blasts BLIMP-1 expression after 5 days of culture with CpG (HD *n* = 9, DS *n* = 10). Differences between groups determined by unpaired Student's *t*-test (^*^p < 0.05; ^**^p < 0.01; ^***^p < 0.001).

To further investigate whether miRs encoded by HSA21 were differently expressed in DS and HD, we studied the basal expression levels of mature miR-155 and miR-125b in PBMCs from HD and DS patients. We found that the expression of miR-155 was significantly higher in DS PBMCs whereas the expression of mature miR-125b was not significantly different between the two groups (Figure 4A). The expression levels of miR-let7c and miR-99a were also comparable between DS and HD (Supplementary Figure S5A).

The function of B cells can be studied *in vitro* by activation with CpG, a synthetic oligonucleotide that binds to TLR9. Whereas naïve B cells show increased survival in response to CpG, MBCs proliferate, and differentiate into PBs (42, 43). We have previously shown that the number of MBCs is severely reduced in the peripheral blood of DS children. In response to CpG, however, MBCs of DS individuals proliferate at a higher rate than the cells from HD and generate a number of PCs that is higher than expected (6). In order to evaluate the expression of miR-155 and miR-125b and their main target genes at both the mRNA and protein level, we studied the B cell response after *in vitro* activation with CpG. miR-155 has been shown to increase in isolated CD19^+^ human B cells stimulated with CpG in patients with rheumatoid arthritis (44). We were unable to purify sufficient numbers of B cells from DS children and for this reason we stimulated total PBMCs with CpG. The kinetics of miR-155 and AICDA expression are shown in Figure 4B. We show that miR-155 was induced by CpG in both HD and DS individuals starting from day 1 and was downregulated at day 3. In PBMCs of DS children miR-155 was significantly more expressed than in the controls at all-time points and remained significantly higher after 5 days of stimulation, when it had returned to baseline in HD. miR-125b was significantly higher in DS, both in unstimulated and stimulated cells (Figure 4C). We also studied the expression of miR-let7c and miR-99a as they are in cluster with miR-125b. Their expression followed the same pattern of miR-125 although let7c is present in very small amounts (Supplementary Figure S5B). Stimulation with CpG significantly upregulated the mRNA expression levels of AICDA and PRDM1 in HD, whereas in DS children both AICDA and PRDM1 were expressed to a lesser extent compared to controls, even though this difference was not statistically significant (Figure 4C). Despite the fact that MBCs of DS children effectively differentiate *in vitro* (6), the frequency of PBs (identified as CD27^++^ CD38^+++^) (43) was significantly lower in DS patients. In *in vitro* derived PBs, the expression level of BLIMP-1, detected by flow cytometry, was significantly reduced in DS children (Figure 4D). Thus, after *in vitro* activation, B cells of DS expressed higher amounts of miR-155 and miR-125b that could explain a dysregulated expression of their target proteins AID and BLIMP-1.

### *In vitro* silencing of miR-155 and miR-125b affected PC formation both in HD and DS

**Figure 5:**
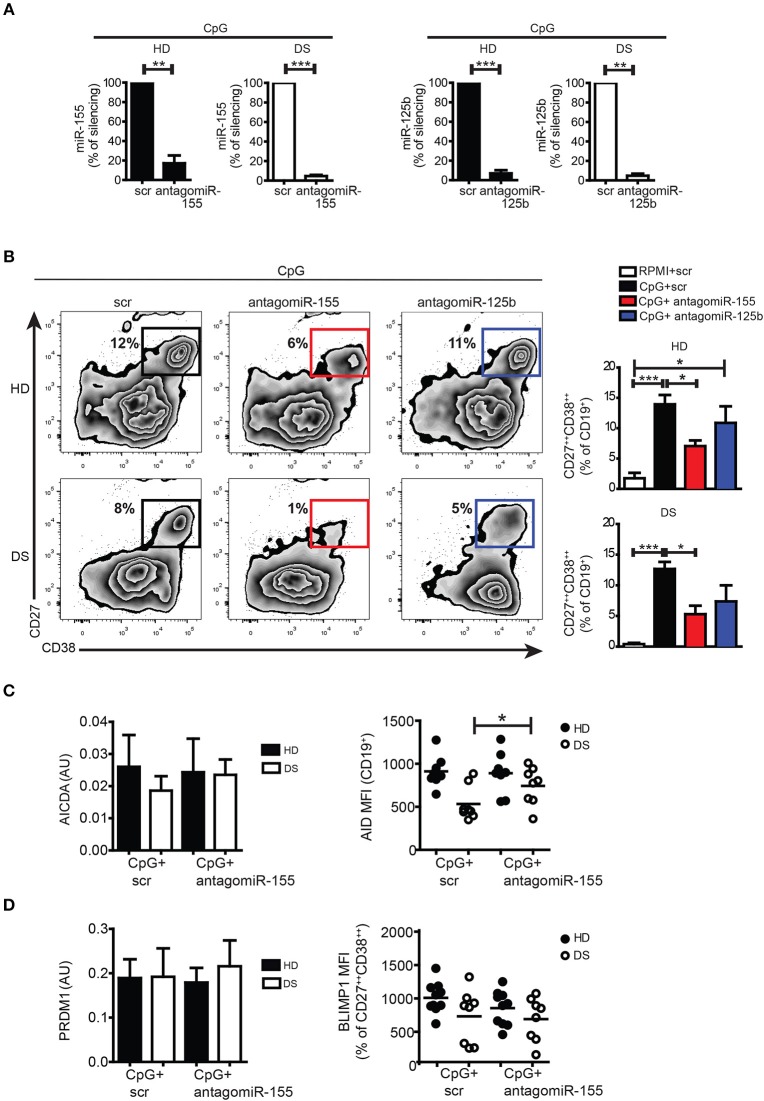
Silencing of mature miRs in activated peripheral blood B cells with CpG. **(A)** PBMCs were treated with antagomiR and activated with CpG for seven days. Graphs show the efficiency of miR-125b and miR-155 silencing in HD (*n* = 4) and DS (*n* = 4) evaluated by qPCR, and expressed as percentage of silencing compared to the scr control. **(B)** After seven days, plasma blasts differentiation was assessed by flow cytometry through the surface upregulation of CD27 and CD38. Plots of a representative HD and DS patient is shown. Bars indicate mean frequency±SEM of plasma blasts for HD (*n* = 14) and DS (*n* = 15). **(C)** Bars show mean±SEM mRNA levels of AICDA in *in vitro* stimulated cells after silencing of miR-155 (right); graph shows MFI levels of AID protein expression evaluated by flow cytometry after *in vitro* silencing of miR-155, each dot represents a different HD or DS, black lines represent mean. **(D)** Bars show mean±SEM mRNA levels of PRDM1 in *in vitro* stimulated cells after silencing of miR-155 (right); graph shows MFI levels of BLIMP-1 protein expression evaluated by flow cytometry after *in vitro* silencing of miR-155, each dot represents a different HD or DS, black lines represent mean. Culture conditions are indicated in figure legend. Differences between groups determined by unpaired Student's *t*-test in A (^**^p < 0.01; ^***^p < 0.001). One-way ANOVA Kruskal-Wallis test followed by Dunn's Multiple comparison test was performed in **(B)** (^*^p < 0.05; ^***^p < 0.001).

Both miR-155 and miR-125b are involved in the GC reaction and PC formation: upregulation of miR-155 can cause premature PC formation by repressing PU.1 and PAX5 (24, 44, 45). miR-155 directly regulates AID (46, 47). miR-125b regulates IRF4 and BLIMP-1 expression (48, 49). In order to verify the function of miR-155 and miR-125b in PC formation, we silenced the mature miRs with antagomiRs in PBMCs of DS and HD, and stimulated them with CpG. After seven days, cells were harvested and stained with appropriate combination of Abs to analyse the differentiation of PBs by flow cytometry. The efficiency of silencing was evaluated by qPCR: we observed around 90% downregulation in the expression of each miR compared to their scramble (scr) antagomiR in both HD and DS (Figure 5A, see section Materials and Methods for details). After treatment with antagomiR-155, differentiation into PBs was significantly reduced both in HD and DS (Figure 5B). Inhibition of miR-125b did not seem to affect PB differentiation (Figure 5B). We also studied the effect of silencing on AICDA and AID. Although at the mRNA level there were no differences between scr and antagomiR-155-treated cells, AID protein was significantly upregulated in DS patients after treatment with antagomiR-155 (Figure 5C). BLIMP-1 was, instead, not influenced by the treatment neither in DS nor in HD (Figure 5D). Thus, inhibition of miR-155 reduces B cell terminal differentiation *in vitro* as demonstrated by the reduced numbers of PCs obtained in cultures containing the miR-155 antagomiR. The inhibition of miR-125b did not impair the generation of PCs *in vitro*.

## Discussion

DS is caused by an extra-copy of HSA21 resulting in a complex dysregulation of genes not only encoded on HSA21 but also on other chromosomes (11). DS individuals suffer from recurrent infections of the respiratory tract and gut and respond poorly to vaccinations (7). Furthermore, they are at high risk of developing autoimmune disorders as well as malignancy (50–52). In normal individuals, MBCs, generated by the immune response to pathogens or vaccines, prevent re-infections. We have shown previously that DS children have a reduced number of MBCs, especially of switched MBCs. *In vitro*, MBCs of DS children show an increased ability to differentiate into PBs (6). The recent discovery of miRs has added a new level of complexity to the study of gene regulation. Each miR can regulate the expression of hundreds of target genes, thus influencing several different pathways and biological processes (53). HSA21 encodes 14 miRs, two of which, miR-155 and miR-125b, play an important role in the immune response (17, 24, 54) and their expression has been found increased in cells of DS individuals. We studied the expression of miR-155 and miR-125b in B cells of DS patients and HD in order to evaluate whether the immunodeficiency associated with DS may be a disorder caused by miRs.

First, we analyzed the GCs where switched MBCs are generated. We show that switched MBCs are significantly reduced in tonsils of DS children. GCs were present in normal numbers, but GCs of DS children were significantly smaller than those of HD. GC B cells and T_FH_ cells, were also significantly diminished in DS compared to HD and the GCs contained less B cells and less T_FH_ cells (Figures 1,2). We then evaluated the expression of miR-155 and miR-125b in sorted cells. The expression of miR-155 was slightly increased in GC B cells of DS children and was significantly higher in tonsil MBCs (Figure 3); miR-125b was significantly increased in both MBCs and PCs. In MBCs of DS children, AID protein was reduced. In T cells miR-155 was overexpressed at the memory T cell stage. The small increase in miR-155 and miR-125b in the GC B cells may be sufficient to impair the fine balance indispensable for the complex events occurring during the immune response.

GC B cells are a very fragile population prone to apoptosis and difficult to manipulate. Thus, we studied miR expression and functions during B-cell activation in a more stable system, using peripheral blood B cells stimulated with CpG *in vitro*. Both miR-155 and miR-125b were increased in B cells of DS children. MBCs proliferated and differentiated in culture. The frequency of PB generated *in vitro* was lower in DS than in the controls probably because of the reduced number of MBCs in the PBMCs (Figure 4 and Supplementary Figure S5C). Interestingly, PB generated *in vitro* expressed reduced levels of BLIMP-1, a phenomenon that could reflect the function of miR-125b.

In order to confirm the functions of miR-155 and miR-125b in B cells, we inhibited their activity in culture with antagomiRs (25, 26). We observed that the inhibition of miR-155 reduced the number of PCs generated *in vitro* in both HD and DS, whereas miR-125b inhibition had no measurable effects on PC differentiation.

miR-155 controls PB formation through the PU.1-PAX5 axis (24, 44, 55). PU.1 maintains the levels of PAX5, which in turn controls B-cell identity and prevents terminal differentiation into PCs. If PU.1 is downregulated by miR-155, PAX5 levels decrease and the PC program is then implemented by the upregulation of BLIMP-1 (44, 54). This mechanism might explain why the few MBCs of DS children show an increased propensity to become PB *in vitro* (6). Accordingly, miR-155 antagomiR reduces the frequency of PBs generated *in vitro* (Figure 5). AntagomiR to miR-125b did not change the number of PBs generated by CpG. It has been recently shown that BLIMP-1 plays a role in the establishment of the PC transcriptome (56), but “once established plasma cell identity is maintained independently of BLIMP-1”. BLIMP-1 is, however, involved in the unfolded protein response allowing secretion and survival of long-lived PCs. The reduced capacity of DS children to maintain the level of specific antibodies after immunization (6) may indicate a reduced number or function of long-lived PCs, but further experiments are necessary to determine whether the increase of miR-125b affects BLIMP-1 and PC longevity *in vivo*.

A similar mechanism may explain the reduction of T_FH_ cells in DS. Recently it has been shown that miR-155 reduces the number of BCL6 positive macrophages in atherosclerotic plaques (57). One interesting possibility is that in T_FH_ cells, similar to B cells, the reduction of BCL6 leads to a premature increase of BLIMP-1 thus inhibiting T_FH_ cells differentiation or survival (58). Our hypothesis is that the increased levels of miR-155 and miR-125b alter B- and probably T-cell functions at multiple levels in DS.

Our data show that the administration of antagomiRs for miR-155 *in vitro* changes the fate of B cells, partially correcting the B cell defects observed in DS, as demonstrated by the increase of AID protein and the reduction of PCs. HSA21-encoded miR have also been shown to control heart development (59), to play a role in leukemia (60), and to act as tumor suppressors (61); furthermore, miR-155 is correlated to dementia in DS (62). As miR activity can be modulated by the administration of antagomiRs, our study opens the way to possible pharmacological therapy not only for immunodeficiency, but also for other clinical aspects of DS, such as leukemia and dementia, where HSA21-encoded miRs may play an important role as well.

## Ethics statement

The study protocols and consent forms were approved by the Ethical Committee of Ospedale Pediatrico Bambino Gesù, Rome, Italy. Informed consent was obtained from parents of children and the study was performed following the guidelines of the Declaration of Helsinki.

## Author contributions

CF, EM, and RC designed research. CF and VM, performed main experiments. EG performed cell sorting. CF, EM, OG, and RC wrote the paper. All authors critically analyzed, discussed and interpreted data, and edited the manuscript.

### Conflict of interest statement

The authors declare that the research was conducted in the absence of any commercial or financial relationships that could be construed as a potential conflict of interest.

## Abbreviations

Ab: antibody
AICDA/AID: Activation Induced Cytidine Deaminase
BCL6: B cell CLL/lymphoma 6
CSR: Class Switch Recombination
DS: Down Syndrome
GC: Germinal Center
HD: Healthy Donor
HSA21: Human Chromosome 21
MBC: Memory B Cell
miR: microRNA
PAX5: Paired box 5
PB: Plasma Blast
PBMC: Peripheral Blood Mononuclear Cell
PC: Plasma Cell
TFH: Follicular Helper T cells
PRDM1/BLIMP-1: PR domain containing 1 with zinc finger domain/ B Lymphocyte-Induced Maturation Protein 1
scr: scramble
SHM: Somatic Hypermutation
Spi1/PU.1: Spi-1 proto-oncogene.
