# Supplementary material for: Dysregulated miR-155 and miR-125b Are Related to Impaired B-cell Responses in Down Syndrome
Source: Front Immunol. 2018 Nov 20;9:2683. doi: 10.3389/fimmu.2018.02683 (PMC6255899; doi:10.3389/fimmu.2018.02683)
Supplement: Supplementary file 1 [file Data_Sheet_1.docx]

**Supplementary Material**

**Dysregulated miR-155 and miR-125b are related to impaired B-cell responses in Down Syndrome**

Chiara Farroni, Emiliano Marasco, Valentina Marcellini, Ezio Giorda, Diletta Valentini, Stefania Petrini, Valentina D’Oria, Marco Pezzullo, Simona Cascioli, Marco Scarsella, Alberto G. Ugazio, Giovanni C. De Vincentiis, Ola Grimsholm and Rita Carsetti^*^

*Correspondence: Dr. Rita Carsetti; [rita.carsetti@opbg.net](mailto:rita.carsetti@opbg.net)


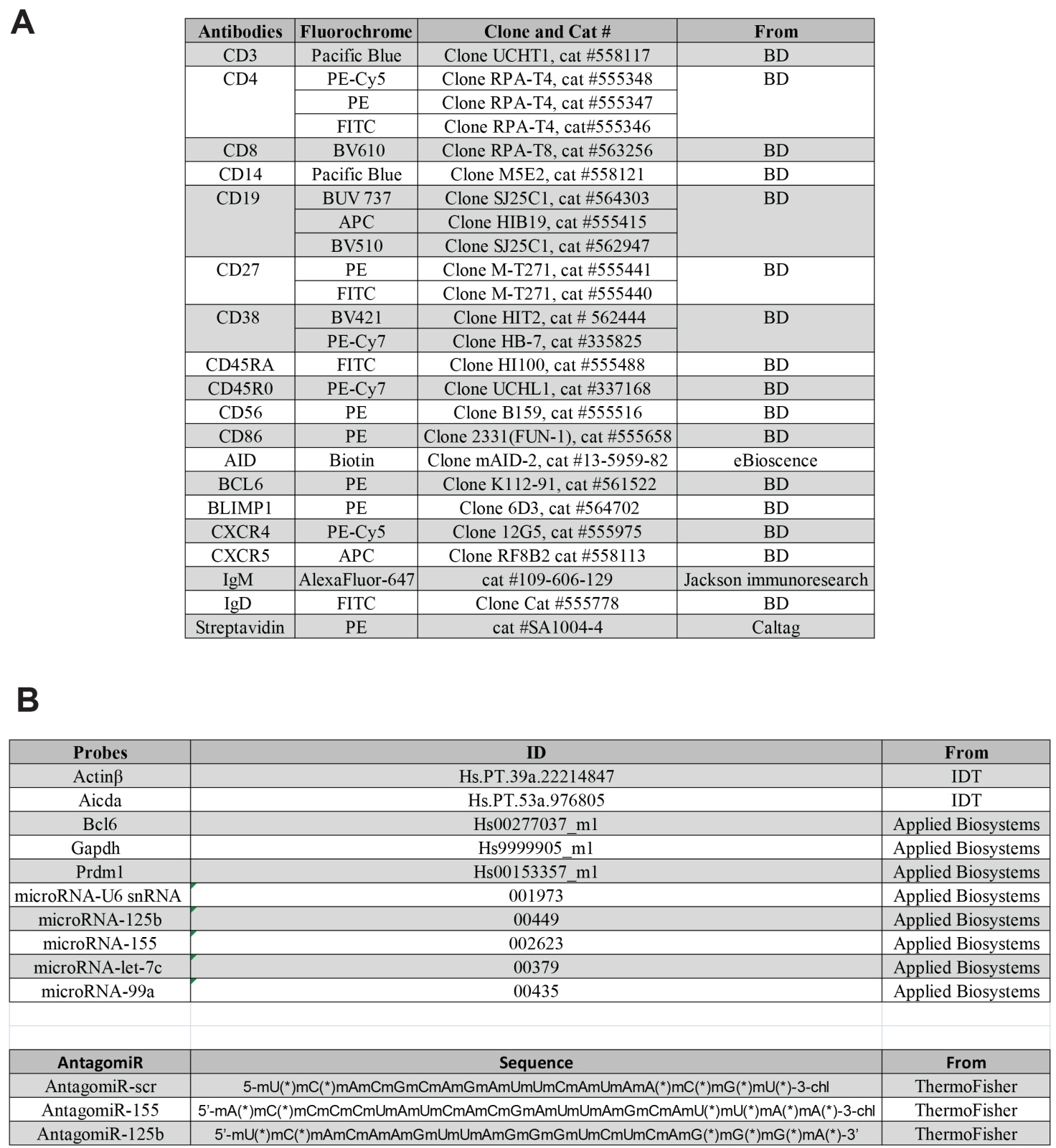


**Supplementary Figure S1.** List of antibodies (A), TaqMan probes and antagomiRs sequences (B).


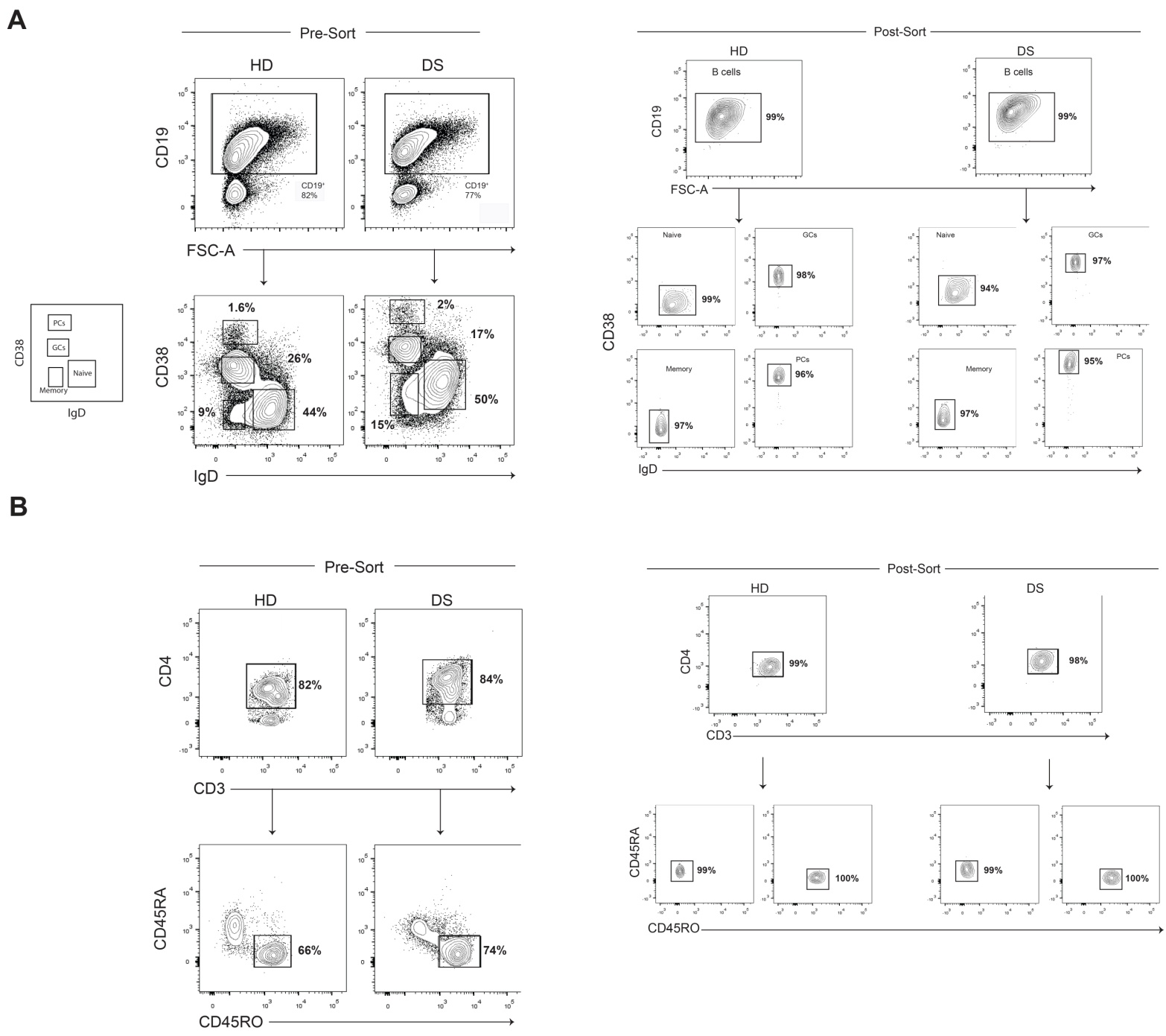


**Supplementary Figure S2.** Gating strategy for sorting tonsillar B-cell subsets (A) and T-cell subsets (B). Post purity of sorted populations is shown.


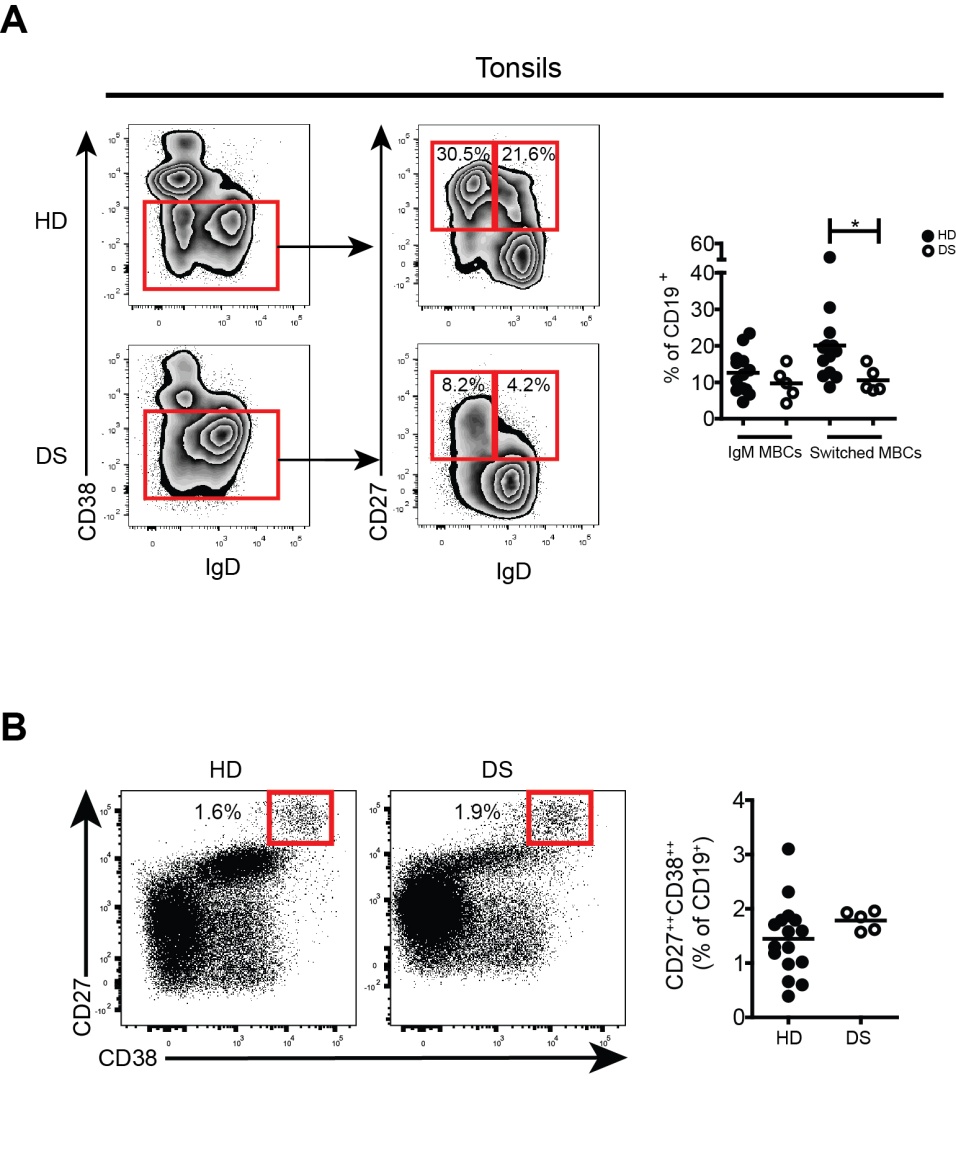


**Supplementary Figure S3. (A)** Plots show the distribution and frequency of IgM and Switched MBCs in tonsils of a representative HD and DS patient. MBCs were identified as IgD^+^ or ^-^ after exclusion of GC and PC (red gate, left panel). Switched MBCs are IgD^-^ CD27^+^, whereas IgM MBCs are IgD^+^ CD27^+^ (right panel). Graph shows the frequency of IgM and Switched MBCs of HDs (n=17) and DS patients (n=5). **(B)** Plots show the distribution and frequency of PCs in tonsils of a representative HD and DS patient, identified as CD38^++^ CD27^++^. Graph shows the frequency of PCs of HDs (n=17) and DS patients (n=5). Differences between groups determined by unpaired Student’s t-test (* p=0.05).

**
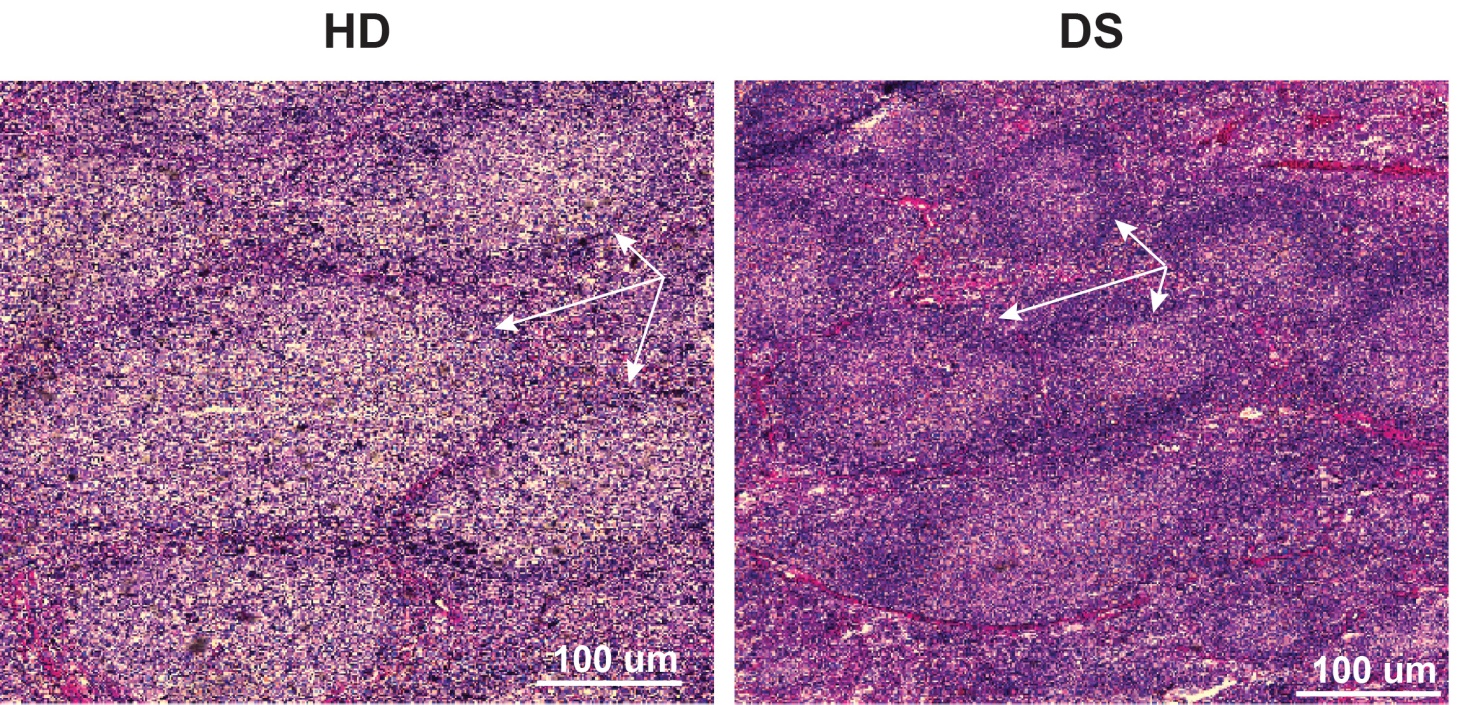
**

**Supplementary Figure S4.** H&E stain of tonsil sections of controls (HD) and patients (DS) is shown. Arrows indicates GCs and scale bars are shown.


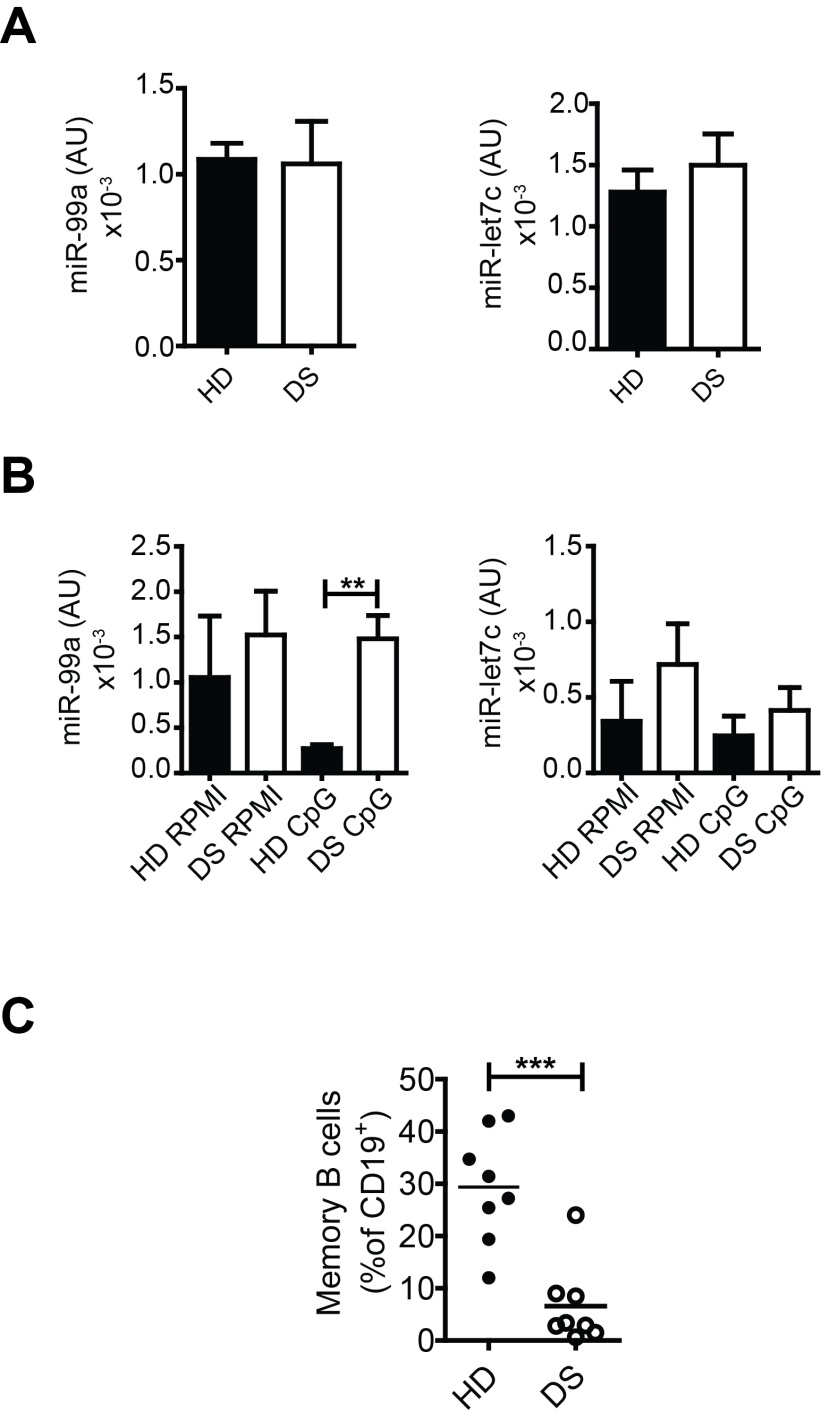


**Supplementary Figure S5.** (A) Bars represent expression of miR-99a and miR-let7c in HD and DS in unstimulated PBMCs. (B) PBMCs of HD and DS were stimulated for five days with CpG, miRs expression was evaluated. (C) Frequency of MBC (gated as percentage of CD19^+^) in the peripheral blood of HD and DS cultured for 7 days in RPMI was evaluated by flow cytometry. Each dot represents a different HD or DS and black lines represent mean. Differences between groups determined by unpaired Student’s t-test (** p=0.01, *** p=0.001).
